# Influence of Pre-Weaning Social Isolation on Post-Weaning Emotion Tendency and Mother–Infant Interactions in Infant *Octodon Degus*

**DOI:** 10.3390/ijerph16101824

**Published:** 2019-05-23

**Authors:** Tomoko Uekita, Akiko Ishibashi, Toshiro Sakamoto

**Affiliations:** Department of Psychology, Kyoto Tachibana University, 34 Yamada-cho, Oyake Yamashina-ku, Kyoto 607-8175, Japan; p1pet2@yahoo.co.jp (A.I.); sakamoto-t@tachibana-u.ac.jp (T.S.)

**Keywords:** *Octodon degus*, social isolation, emotionality, infant–mother interaction

## Abstract

Our previous research using *Octodon degus* (degus) revealed that preweaning social isolation negatively affected object exploratory behavior. However, it remains unknown how social isolation affects animal psychology and other behaviors. The present study examined the effects of neonatal social isolation on degu emotion and mother–infant interactions before and after weaning. Because degus have a complex social repertoire, we predicted that they would be sensitive to social isolation and show similarities with humans in their social interaction. Pups in the isolation group were separated from their family seven times for 30 min a day from 8 to 15 days post-birth. Pups in the nonisolation group were reared with their family. At 2, 3, 4, 5, and 6 weeks of age, pups underwent a zero-maze test to measure anxiety and a mother–infant interaction test to assess mother–infant attachment. Isolated pups showed more activity in the zero-maze test than nonisolated pups at 3 weeks of age. We found no significant effects of social isolation on mother–infant interactions. These results suggest that while neonatal social isolation might affect emotion during weaning, it does not influence mother–infant relationships.

## 1. Introduction

**Figure 1:**
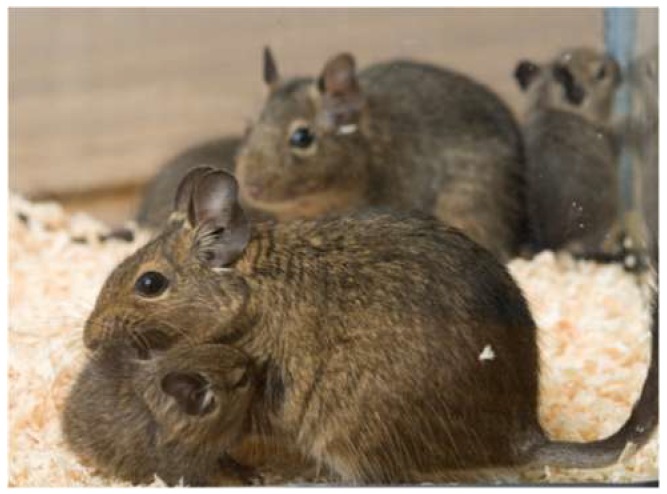
A degu family. Mother and her pup (front) and father with siblings (back).

In the formative attachment period, decreased communication between a child and his/her mother can negatively influence the child’s growth. For example, Bowlby reported that children who received maternal deprivation showed a delay in physical growth and psychological development [1,2]. In the last two decades, *Octodon degus* (degus, Figure 1) have been used as an animal model for the study of attachment, with which researchers have investigated the effects of maternal deprivation or social isolation on the central nervous system and behavior. Degus are often used in attachment studies because they display several relevant social traits. For example, they live in extended family groups consisting of one or two males and one to four females and their young [3,4], they cooperate to increase vigilance to predators [5,6,7] and communicate with each other using more than 15 types of sounds [8,9]. These social traits could indicate that degus are sensitive to social environments, and that they might show human-like behavioral changes in response to social isolation.

Table 1 summarizes previous social isolation studies in degu pups. In terms of social isolation and the brain, Braun’s research group concluded that maternal separation and social deprivation during the early phase of life can affect pup neurophysiology. In their study, degu pups in an experimental group were repeatedly separated from their mothers from P1 (postnatal day 1) to P21 and were then reared in complete isolation after weaning until P45. Pups in the control group were reared under normal social conditions until P45. Pups in the experimental group showed a substantial decrease in the number of nicotinamide adenine dinucleotide phosphate- diaphorase- reactive (NADR) neurons in the dorsal anterior cingulate cortex and nucleus accumbens [10]. Additionally, in a follow-up study, the author showed that social separation altered the balance between serotonergic and dopaminergic innervation of the medial prefrontal cortex [11].

Because of the experimental design, the studies from Braun et al. could not clarify whether preweaning maternal separation or postweaning social isolation had the greater influence on neural development, [10,11]. In degus, normal weaning begins at about 3 weeks of age [12]. Degu pups need 4 or 5 weeks for complete weaning, while taking in both solid food and milk afterwards [13]. In a subsequent study, the Braun research group reported that preweaning parental separation (from P1 to P21) altered the spine densities in several brain areas. The spine densities of the dendrites in the cingulate cortex and hippocampal CA1 region increased, whereas those in hippocampal dentate gyrus and amygdala decreased [14]. The Braun research group also reported that shorter-term social separation from mothers and siblings during preweaning (from P8 to P10, 3 min, twice per day) resulted in an increase in dopaminergic and serotonergic receptor density in the CA1 region of the hippocampus [15]. These results indicated that even transient separation has a large impact on the developing limbic system when it occurs in the very early days after birth.

In addition to its effect on the brain, researchers have also examined how isolation affects the future behavior of degu pups. Braun et al. (2003) investigated whether preweaning social separation would change novelty-induced exploratory behavior in an open field test [12]. The pups were placed in isolation cages for 1 h, three times per day from P1 to P7. On P8, their activity was measured individually with a 3-min trial in an open field test, and results showed that isolated pups engaged in running activity ran significantly more than nonisolated pups. Thus, parental separation very early in life enhanced degu locomotor activity in an unfamiliar environment.

Colonnello and colleagues (2011) assessed whether social isolation after achieving mother recognition alters degu pup locomotor activity in novel environments and approach behavior toward the mother [16]. It takes more than 2 weeks for degu pups to establish mother recognition [18]. The aforementioned study by Braun et al. (2003) assessed the isolation effect before establishing mother recognition [12]. To assess the isolation-effect on the stage of mother recognition, Colonnello et al. divided degu pups into three groups (social, restricted, and isolated) on P21, and reared them with varying degrees of social contact for 10 days. The social group was housed with family members (Group A). The restricted group was kept in isolation (Group B), but was allowed to interact with family members 30 min/day through a wire mesh. The isolated group was placed in continuous isolation (Group C). After 10 days, all pups completed two tests once per day for 5 days. The first test was a 10-min open field test in which their activity was recorded. The second test was 10-min social choice test in which preference for their mothers over unfamiliar females was recorded. The isolated degus exhibited significantly greater locomotor activity and defecation in the open field test. Colonnello et al. interpreted these results as a greater desire to escape from the environment, which was related to social isolation-induced anxiety. In the social choice test, nonisolated degus spent more time close to their mothers at the beginning of the test (i.e., they preferred their mothers), but this time decreased as the test was repeated. According to Colonnello et al., this gradually reduced contact with their mothers is similar to what is seen in human children who are secure in their attachment to their mothers. In contrast, the isolated degus had no preference for their mothers or for unfamiliar females, which indicates that social isolation had prevented them from forming attachments to their mothers.

Uekita and Kawakami (2016) investigated how the isolation experienced before weaning influenced exploratory behavior after weaning [17]. They isolated pups from their family for 30 min a day, 14 times from P6 to P23. Nonisolated pups were reared with their families. Then at 3–7 weeks of age, they assessed exploratory behavior directed toward a novel object inserted into their home cage under the two following conditions: with-mother and without-mother. At 3 weeks of age, the nonisolated pups more frequently contacted the novel object when their mothers were present than when they were not. However, those in the isolated group did not explore actively even when their mothers were present and showed no difference in the frequency of contact between the two conditions. These results indicated that repetitive social separation in early life negatively affected exploratory behavior, and the authors proposed that isolation experience modified the formation of the mother–pup emotional bond such that the mothers did not function as a secure base.

The aim of the current study was to test the hypothesis that anxiety levels would increase as a result of social isolation, and it would be caused by the modification of mother-pup emotional bond. We examined how early social isolation after pups could recognize their mother influenced two factors: pup emotional tendencies (anxiety) and infant–mother interaction (IMI). For evaluating emotional tendency, we used an elevated zero maze (EZM). The EZM has open quadrants and closed quadrants with walls. For rodents, the open quadrants are perceived as the dangerous areas, whereas the closed quadrants are perceived safer [19]. If early social isolation increases anxiety level, then isolated pups should stay longer in the closed quadrants. To assess the influence that social isolation has on IMI, we measured approaches from the infant to mother and mother to the infant in a novel environment.

## 2. Materials and Methods

All experiments and animal care were conducted in accordance with the Kyoto Tachibana University guidelines for animal experiments. Protocol was approved by the Ethics Committee for Animal Experiments of Kyoto Tachibana University (Code: 17-02).

### 2.1. Animals

We used ten degu pups born to two couples. One family group included three male pups and two female pups, and the other included two male pups and three female pups. Their mothers were also used in the experiments. At the beginning of the behavioral test (pups were 2 weeks of age), the mean body weights were 26.6 ± 1.02 g for the nonisolated group and 26.4 ± 1.50 g for the isolated group. Body weights for the mothers were 208 g and 210 g. The home cage was 35 cm × 60 cm × 45 cm (W × D × H), and it contained a wooden nest, a water bottle, and a food cup. The animals were bred by a family unit, which comprises an adult couple and their offspring. The father was removed from the home cage at the last day of the isolation period. The vivarium was maintained at 23 ± 2 °C and humidity was 70% or less, with a 12-h light–dark cycle starting at 7 a.m. Food (Brisky, NY, USA) and water were available ad libitum in the home cage. At the beginning of the experiment, part of each pup’s tail was colored using odorless nail polish for identification.

### 2.2. Apparatus and Materials

**Figure 2:**
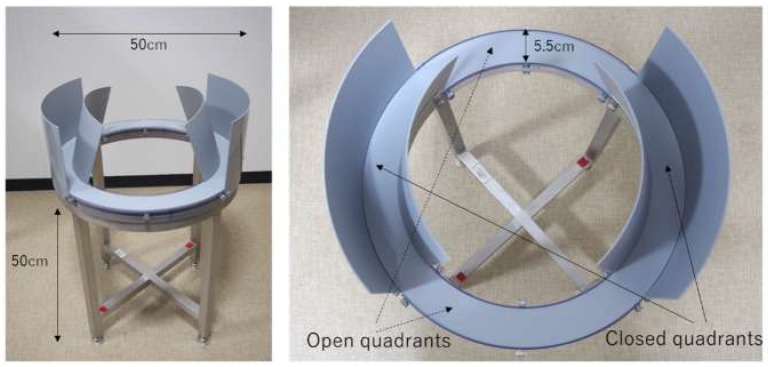
The elevated zero maze (EZM) seen from the side (**left**) and from above (**right**).

For isolation, we used a newly cleaned cage (28 cm × 45 cm × 20 cm) with a stainless steel-wire cover for each pup. To measure emotional tendency, we used an EZM (O’Hara, Tokyo, Japan), which was set in the middle of the experimental room. The annular platform (50 cm diameter; 5.5 cm height) was placed 50 cm above the floor. The platform has two enclosed quadrants with 18-cm-high walls and two open quadrants (Figure 2). A video camera (GV-HD700, SONY, Tokyo, Japan) was installed in the ceiling above the EZM to record the data.

The IMI test was conducted in a sound-insulated box (107 cm × 60 cm × 60 cm) that had a black board on one side of a wall and an LED light on the ceiling. The brightness of the inside of the sound-insulated box was 118.8 lx. Two CCD cameras (Watec, Yamagata, Japan) on the ceiling were connected to a digital video recorder. We observed animal behavior through a monitor.

### 2.3. Behavioral Procedure

#### 2.3.1. Separation from Parents and Siblings

Pups were designated to either the isolated or nonisolated group (*n* = 5 for each) according to body weights at P9 so that the mean body weights of two groups were equal. Both groups contained pups from both families. There were two males and three females in the isolated group and three males and two females in the nonisolated group. The separation procedure was the same as that described by Uekita and Kawakami (2016) [17], except for the start time and the number of repetitions of separation. Degus in the isolated group were separated from their family once per day from P8 until P15 (seven times in total). Body weight was measured before separation each day. During the separation, animals were removed from their home cage and placed alone in an isolation cage for 30 min. The isolation cages were placed next to each other in the vivarium. The pups in the isolated group could see and hear their siblings, each of whom was placed in a separation cage different from that of the isolated pups. The parent degus remained in the home cage during the separation period and could not be seen by the isolated pups.

#### 2.3.2. Behavioral Experiments

Following separation from parents and siblings, pups completed the EZM and IMI tests once per week from 2 to 6 weeks of age (five times each; P16, P24, P29, P37, and P44). Both tests lasted 10 min and were performed in succession (EZM followed by IMI). Body weights were measured each day before the tests.

The EZM test: Prior to the EZM test, an experiment subject was removed from the home cage to the waiting cage used for separation. At the start of the test, a degus pup was placed on one of the closed quadrants and allowed to explore 10 min. The test scene was video recorded for off-line analysis. As the parameters of emotionality and locomotor activity, we measured the time spent in closed quadrants, the number of entries into closed quadrants from open quadrants, and the number of entries into open quadrants from closed quadrants. When the two forepaws entered the open/closed quadrants, this was counted as an entry.

The IMI test: The mother and a degu pup were put in separate waiting cages and placed in front of the sound-insulated box. We placed the mother in the sound-insulated box first, then placed the pup inside 1 min later. Then, the two degus were allowed to interact for 10 min. The test was video recorded for off-line analysis using Behavior Coding System 2 (BECO2; Ltd. DKH, Tokyo, Japan). We recorded behavior of both pup and the mother and recorded the time and frequency at which they approached each other.

#### 2.3.3. Statistical Analysis

For both the EZM and the IMI tests, we divided the 10-min test period into two 5-min epochs to evaluate changes in behavior within a trial. We used a three-way analysis of variance (ANOVA) with “group” (isolated or nonisolated) as a between-subject factor, and “age” (2, 3, 4, 5, or 6 weeks) and “trial epoch” (first half or second half) as within-subject factors. Ryan’s method was used for multiple comparisons when the effect of age was significant. Changes in body weight were analyzed using a two-way ANOVA with “group” (isolated or nonisolated) as a between-subject factor and “age” (postnatal day or weeks of age) as a within-subject factor.

## 3. Results

### 3.1. The EZM Test

**Figure 3:**
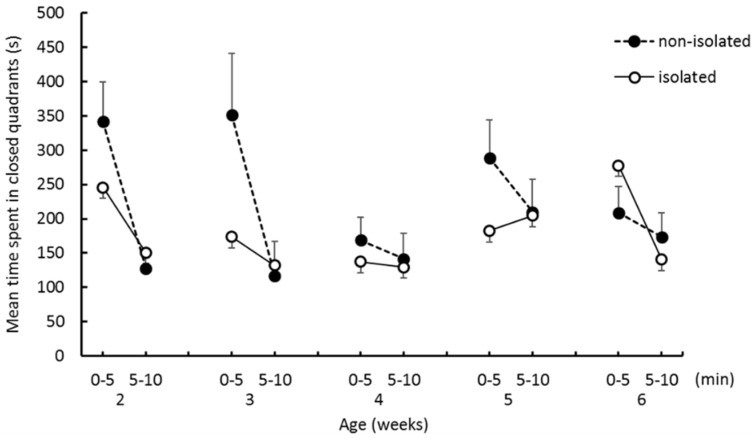
The mean time spent in the closed quadrants during the EZM test. Error bars indicate the standard error.

Figure 3 shows the mean time spent in closed quadrants for both groups. Although the mean times spent in closed quadrants were longer in the nonisolated group than the isolated group during the first half of trials at 2, 3, and 5 weeks of age, these differences were not significant. The time spent in closed quadrants decreased from the first to second half of trials in the nonisolated group, so that it became similar to that of the isolated group. The three-way ANOVA revealed significant main effects of “age” (*F*_(4,32)_ = 2.96, *p* < 0.05) and “trial epoch” (*F*_(1,8)_ = 12.21, *p* < 0.01). Ryan’s method showed significant age-related differences between time spent in closed quadrants at 4 weeks and that at 5 weeks (*p* < 0.05).

**Figure 4:**
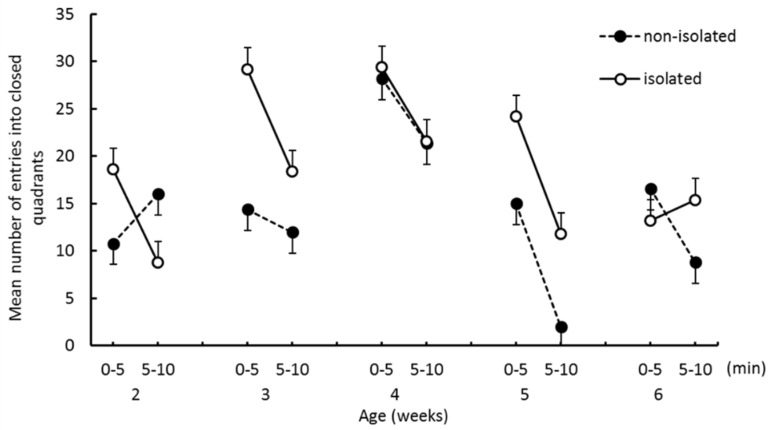
The mean number of entries into closed quadrants during the EZM test. Error bars indicate the standard error.

Figure 4 shows the mean number of entries into closed quadrants from open quadrants. Although entries into closed quadrants were more frequent in the isolated group at 3 weeks, the differences were not significant. The mean number decreased in the second half of trials for all weeks except at 2 weeks for the nonisolated group and at 6 weeks for the isolated group. A three-way ANOVA revealed a significant main effect of “trial epoch” (*F*_(1,8)_ = 46.79, *p* < 0.001). We also found a significant first-order interaction between “age” and “trial epoch” (*F*_(4,32)_ = 3.00, *p* < 0.05) and a second-order interaction among “group”, “age”, and “trial epoch” (*F*_(4,32)_ = 3.83, *p* < 0.05). We also found significant simple interactions between “group” and “trial epoch” at 2 weeks and 6 weeks (*p* < 0.05). The simple-simple main effects of “trial epoch” were significant at 4 weeks (*p* < 0.05), 5 weeks (*p* < 0.001), and 6 weeks (*p* < 0.05) for the nonisolated group, whereas they were significant at 2 weeks (*p* < 0.01), 3 weeks (*p* < 0.005), 4 weeks (*p* < 0.05), and 5 weeks (*p* < 0.001) for the isolated group.

**Figure 5:**
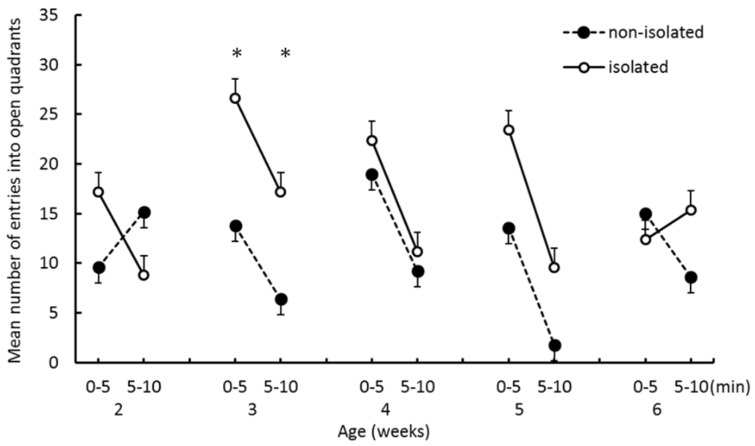
The mean number of entries into open quadrants during the EZM test. Error bars indicate the standard error. *significance at *p* < 0.05.

Figure 5 shows the mean number of entries into open quadrants from closed quadrants. Entries into open quadrants were more frequent in the isolated group than in the nonisolated group at 3 weeks. The number of entries lessened from the first half to the second half of trials for both groups, except for the nonisolated group at 2 weeks and isolated group at 6 weeks. A three-way ANOVA revealed a significant main effect of “trial epoch” (*F*_(1,8)_ = 34.71, *p* < 0.001). We found a significant first-order interaction between “age” and “trial epoch” (*F*_(4,32)_ = 5.28, *p* < 0.005) and a second-order interaction among “group”, “age”, and “trial epoch” (*F*_(4,32)_ = 3.38, *p* < 0.05). We also found significant simple interactions between “group” and “trial epoch” at 2 weeks (*F*_(1,40)_ = 8.98, *p* < 0.005). The simple-simple main effects of “group” were significant at the first half and second half of trials at 3 weeks (*p* < 0.05).

### 3.2. The IMI Test

**Figure 6:**
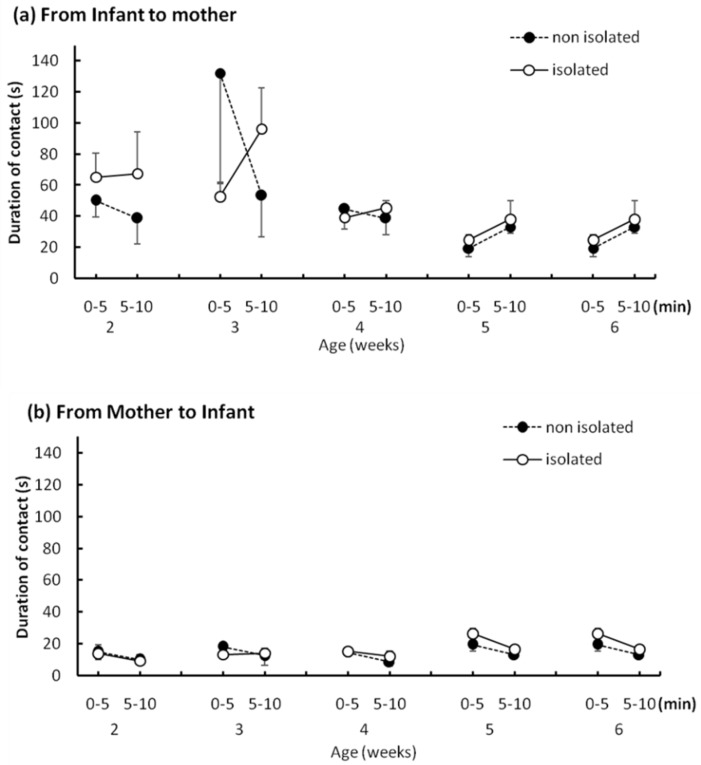
The mean duration of contact initiated by infant to mother (**a**) and mother to infant (**b**). Error bars indicate the standard error.

Figure 6a shows the duration of social contact that pups directed to their mothers. The duration of contact in the first half of the trial was longer for pups in the nonisolated group at 3 weeks than at 2 weeks. However, contact duration decreased in the second half of the trial at 3 weeks for the nonisolated group. A three-way ANOVA revealed a significant main effect of “age” (*F*_(4,32)_ = 5.15, *p* < 0.005). Ryan’s method showed significant age-related differences in mother-directed contact duration at 3 weeks and that at 4 weeks, 5 weeks, and 6 weeks (*p* < 0.05). There were no between-group differences in the duration of social contact that mothers directed to their pups (Figure 6b). We found no between-group difference. A three-way ANOVA revealed a significant main effect of “age” (*F*_(1,32)_ = 9.56, *p* < 0.05).

**Figure 7:**
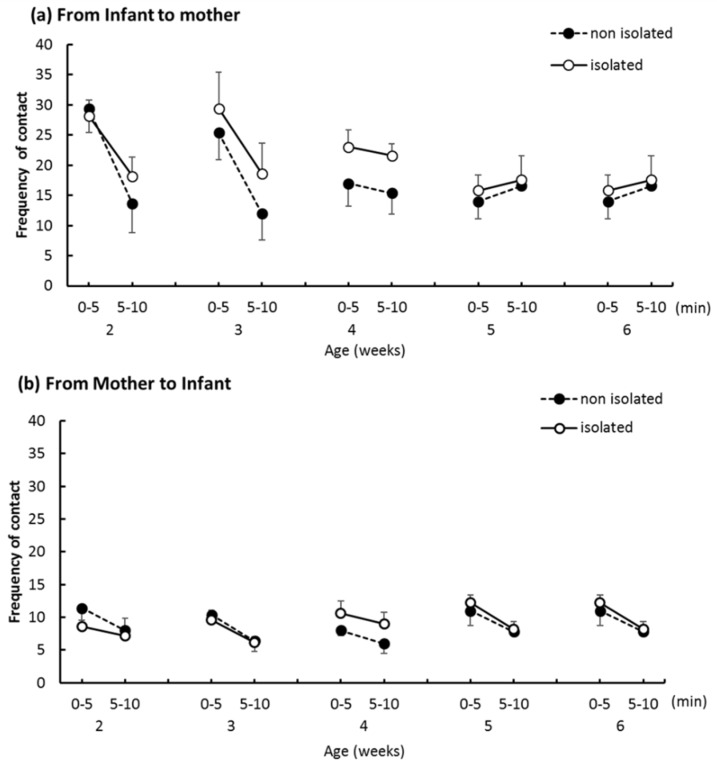
The mean frequency of contact initiated from infant to mother (**a**) and mother to infant (**b**). Error bars indicate the standard error.

Figure 7a shows the frequency with which pups initiated contact with their mothers. A three-way ANOVA revealed a significant main effect of “trial epoch” (*F*_(1,32)_ = 21.35, *p* < 0.005) and a first-order interaction between “age” and “trial epoch” (*F*_(4,32)_ = 13.45, *p* < 0.001). At 2 weeks and 3 weeks, contact frequency significantly decreased from the first half to second half of trials. There were no significant between-group differences in the frequency with which mothers initiated social contact with their pups (Figure 7b). A three-way ANOVA revealed a significant reduction in the number of pup-directed contact from the first to second half of trials (*F*_(1,32)_ = 13.99, *p* < 0.01).

**Figure 8:**
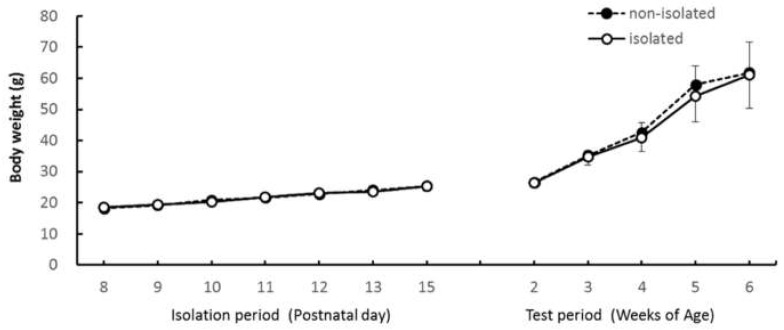
The mean body weight of the isolated and nonisolated groups during the isolation period (**left**) and the test period (**right**). Error bars indicate the standard error.

We found no significant between-group differences in body weight changes during the isolation period nor the test period (Figure 8); body weight of both groups increased equally with age. A two-way ANOVA showed main effects of “age” in the isolation period (*F*_(6,48)_ = 171.38, *p* < 0.001) and in the test period (*F*_(4,32)_ = 103.02, *p* < 0.001). Ryan’s method showed significant differences in body weight between all age points (*p* < 0.05) within both periods.

## 4. Discussion

The purpose of this study was to investigate the effects of social isolation occurring before 2 weeks of age on emotional tendencies and infant–mother interaction in degu pups. The effects of isolation were evident at the initial stage of weaning. One of our hypotheses was that anxiety levels would increase as a result of social isolation, which would manifest as spending longer in the closed quadrants of the EZM. We found that pups in the isolated group entered open quadrants more frequently than pups in the nonisolated group, and that the time spent in closed quadrants did not significantly differ between groups. Thus, our EZM results do not support the hypothesis. Furthermore, the interaction effects between “group” and “trial epoch” were significant on both the number of entries into closed quadrants and the number of entries into open quadrants at 2 weeks of age. Isolated pups more frequently entered both quadrants in the first half than in the second half. Nonisolated pups entered quadrants less frequently in the first half than in the second half. Thus, when nonisolated pups were put in the novel environment, they stayed in the closed area for a while before starting to explore the other quadrants, whereas the isolated group frequently moved from closed to open areas from the start of the trial. These results suggest that social isolation enhanced the pup’s locomotor activity and drive to explore open areas at the initial stage of weaning.

In a previous study, we compared object exploration behavior between isolated and nonisolated pups [17]. The isolated pups took longer to come out from their nest, and even with their mother present in the test environment, they explored novel objects less frequently than nonisolated pups. Such social isolation-induced inhibition of exploration was not shown in our present study. This discrepancy might have resulted from differences in the procedures between the two studies. The major procedural difference was the isolation period. In our previous study, isolated pups were separated from their family 14 times for 30 min/day from P6 to P23, while in present study, pups were separated seven times for 30 min/day from P8 until P15. Thus, the short-term isolation of the current study did not suppress exploration, but rather facilitated exploration of open areas. The test environment also differed between the two studies. In our previous study, degu pups were allowed to explore novel objects put in their own home cages. In contrast, degu pups in the present study allowed to explore an unfamiliar environment without any concrete objects. These environmental differences could have led to different levels of excitement, which then altered the effects of social isolation on exploratory behavior.

Previous open-field studies have reported that social isolation causes hyperactivity [12,16]. This apparatus in these studies had no closed spaces that could function as shelters. The authors interpreted hyperactivity observed in this test as being caused by heightened anxiety, possibly reflecting attempts to escape from the open area. According to this interpretation, isolated pups should stay longer in the closed quadrants of the EZM than in the open quadrants. Because this was not supported by our results, we are cautious in interpreting increased activity after social isolation as increased anxiety The greater locomotor activity in the social isolation group can instead be interpreted as reflecting a diminished ability to predict danger in potentially hazardous environments as a result of early isolation.

Our findings that early isolation affected behavior at the initial stage of weaning, especially at 3 weeks, were consistent with our previous study [17] despite the different time points at which isolation began and regardless of how many times isolation was performed. Thus, it seems that the third week of life is a critical stage for observing the effects of early isolation on exploration behavior. Degu pups require suckling for at least 3 weeks to gain enough nutrients and immunity. Before weaning, they start to eat solid food, but cannot digest this until they are about 15 days old [12,20]. We started the isolation protocol when pups were 8 days old and repeated it until pups were 15 days old. Separation from the mother before weaning can have a critical effect on pup survival. The 30-min preweaning separation from the mother in our experiments was thought to be very stressful for the pups. The effects of such stressful events might have surfaced at 3 weeks of age when their behavioral repertoire increases.

We hypothesized that social isolation might affect the mother–infant relationship. However, the IMI test revealed no difference in the time or frequency of direct mother-to-pup contacts. This indicates that the mothers’ attitudes toward pups did not change when the pup was separated from the family for short intervals each day. Additionally, body weights of the two groups were comparable and increased at the normal rate for captive bred degus [21], which indicates no malnutrition or neglect by the mother. In our study, the pup-to-mother directed approaches did not differ significantly. A previous study reported that isolated rats showed an increased frequency and decreased duration of contact with novel partners [22]; this was interpreted to mean that isolated reared rats are interested in partners but cannot continue contact behavior with them for extended amounts of time. While the partner in the present experiment was a mother and not an unfamiliar individual, if a novel partner were introduced into a novel environment, we would expect isolated pups to show more approach behaviors in their social communication efforts.

## 5. Conclusions

The present results indicated that repetitive short-term separation from family in the early postnatal period has an effect on the behavior on EZM in weaning degu offspring. We showed that this was not necessarily brought about by a change in the mother–offspring relationship, but might be related to increased activity or suppressed excitement in the test environment. However, the increased activity on EZM did not continue constantly during the trial. The number of entries into open quadrants reduced from first to second half of trials in isolated pups. Therefore, the isolated pups could also suppress the exploratory behavior that was raised by a novel environment.

In human attachment research, it is thought that the ability to perceive and express emotions is formed through attachment relationships. Emotional arousal more easily occurs thorough experience, after which particular emotional patterns called emotional biases are formed. These emotional biases are formed depending on the interaction with the caregiver [23]. In our study, the emotional alteration shown in the increase in activity was limited to the early stage of weaning, which could be because the mother’s contact and rearing attitude did not change according to changes in the offspring’s activity.

This study found a causal relationship between social isolation and emotionality of offspring. Further studies are needed to investigate the degree to which a novel environment influences future behavior in the parent–offspring relationships in isolated degus.

## Figures and Tables

**Table 1:** Summary of previous studies on the effects of social isolation.

Author	Period of Social Isolation	Brain Area or Behavioral Test	Results
		Brain area	
Poeggel et al. 1999 [10]	P1–P45 (P1-P21 1 h × 3 times/day, P22–P45 24 h)	Cingulate cortex, Nucleus accumbens	number of NADR neuron ↓
Braun et al. 2000 [11]	P1–P45 (P1-P21 1h × 3 times/day, P22–P45 24 h)	Medial prefrontal cortex	alteration of the balance between serotonergic and dopaminergic innervation
Poeggel et al. 2003 [14]	P1–P21 (1 h/day)	Cingulate cortex, Hippocampal CA1 Dentate gyrus, Amygdala	Spine density ↑ Spine density ↓
Ziabreva et al. 2003 [15]	P8–P10 (3 min × 2 times/day)	Hippocampal CA1	dopaminergic and serotonergic receptor density ↑
		Behavioral test	
Braun et al. 2003 [12]	P1–P7 (1 h × 3 times/day)	Open field test	activity ↑, vocalization ↓, response to maternal call ↓
Colonnello et al. 2011 [16]	P21–P30 Group A: no isolation Group B: restricted interaction (30 min/day) Group C: complete isolation	Open field test, Social choice test (10 min × 5 days)	activity ↑ A: preference to mother ↑ (secure attachment) B: preference to mother ↑↑ (insecure attachment) C: preference to mother ↓ (no social preference)
Uekita & Kawakami 2016 [17]	P6–P23 (30 min/day × 14 days)	Object exploration, (5 times, from 3 weeks to 7 weeks)	object exploration with mother at 3rd week↓ start latency at 3rd week↑

Arrows indicate the direction of changes in cases of social isolation. P, Postnatal day.
